# Father–Child Longitudinal Relationship: Parental Monitoring and Internet Gaming Disorder in Chinese Adolescents

**DOI:** 10.3389/fpsyg.2018.00095

**Published:** 2018-02-06

**Authors:** Binyuan Su, Chengfu Yu, Wei Zhang, Qin Su, Jianjun Zhu, Yanping Jiang

**Affiliations:** ^1^School of Psychology, Psychological Counseling & Research Center, South China Normal University, Guangzhou, China; ^2^School of Education, Guangzhou University, Guangzhou, China; ^3^Guangdong Polytechnic of Science and Technology, Guangzhou, China; ^4^Department of Health Promotion, Education, and Behavior, University of South Carolina, Columbia, SC, United States

**Keywords:** parental monitoring, Internet gaming disorder, mother–child relationship, father–child relationship, adolescents

## Abstract

Although empirical studies have indicated that parents have an important role in preventing Internet gaming disorder in adolescents, longitudinal research on the parental predictors of Internet gaming disorder is lacking. We used a three-wave cross-lagged panel model to explore the reciprocal association between parental monitoring and Internet gaming disorder, and examined the different impacts of mother– and father–child relationships on this association. A sample of 1490 adolescents aged 10–15 years (*M* = 12.03, *SD* = 1.59; 45.4% female) completed assessments at all three points. The cross-lagged model revealed that (a) parental monitoring at T1 predicted lower Internet gaming disorder at T2, and greater Internet gaming disorder at T2 predicted lower parental monitoring at T3; (b) father–child relationship had a reciprocal, indirect effect on the relationship between parental monitoring and Internet gaming disorder, while mother–child relationship did not. These findings suggest that the parental effects (e.g., higher parental monitoring and better father–child relationship) might play a vital role in preventing Internet gaming disorder in adolescents.

## Introduction

Internet gaming disorder in adolescents is becoming a widespread problem, particularly in China. According to the Fifth edition of the Diagnostic and Statistical Manual of Mental Disorders (DSM-5), Internet gaming disorder can be defined as “Persistent and recurrent use of the Internet to engage in games, often with other players, leading to clinically significant impairment or distress” (American Psychiatric Association, 2013). Overall prevalence rates of Internet gaming disorder range from 0.7 to 27.5%, depending on the sample (Mihara and Higuchi, 2017). The prevalence rates among adolescents have been researched more extensively, and range from 1.2 to 5.0% in European samples and 7.5 to 26.7% in Asian samples (e.g., Lopez-Fernandez et al., 2013; Yu et al., 2015; Feng et al., 2017; Laconi et al., 2017; Mihara and Higuchi, 2017). In Europe, more than 90% of adolescents surf the Internet daily, and almost all adolescents are active Internet users (Symons et al., 2017). Research on Internet surfing behavior among Chinese adolescents has revealed that among the 287 million (85.3% in total) adolescent Internet users, 66.3% of primary school students and 70.0% of middle school students played online games. Furthermore, 90% of all users used their smartphones to surf the Internet at home (China Internet Network Information Center, 2016). Online games are particularly attractive to youth, and are associated with a greater risk of Internet gambling addiction in youths compared to adults (Young and de Abreu, 2011; Griffiths et al., 2015). Internet gaming disorder in adolescents might not only decrease their emotional intelligence, impair interpersonal relationships, and lead to academic and behavioral problems, but also might be a significant risk factor of anxiety, depression, social dysfunction, and suicidal ideation and behaviors (Przybylski et al., 2010; Young and de Abreu, 2011; Li et al., 2014; Griffiths et al., 2015; Mihara and Higuchi, 2017).

Parental monitoring is defined as parents’ practices and knowledge concerning their children’s activities, whereabouts, and peers (Stattin and Kerr, 2000; Borawski et al., 2003; Bleakley et al., 2016). In keeping with the expansion of Internet use at home and during leisure time in adolescents (China Internet Network Information Center, 2016), a growing body of empirical research has examined the relationship between parental monitoring (along with other parental factors) and Internet gaming disorder. Recent studies have highlighted that low parental monitoring and poor parent–child relationship predicted higher levels of Internet gaming disorder in adolescents (Liu et al., 2012; Koo and Kwon, 2014; Li et al., 2014; Bleakley et al., 2016; Jang and Ryu, 2016). However, the majority of research has focused on unidirectional effects of these two variables on Internet gaming disorder in adolescents; to our knowledge, no studies have yet examined the reciprocal relationships between parental monitoring, parent–child relationship, and Internet gaming disorder during adolescence. Furthermore, few longitudinal studies have explored the mediating effects of relationship of the different parents on the association between parental monitoring and Internet gaming disorder. Thus, we pursued these topics in the present study.

### Parental Monitoring and Internet Gaming Disorder

Some empirical studies have suggested that parental monitoring can reduce the risk of problematic behaviors such as problematic Internet use, substance use, risky sexual behavior, and delinquency (Liau et al., 2008; Xu et al., 2012; Bleakley et al., 2016). In recent years, society has witnessed a massive increase in Internet use in family life (e.g., Álvarez et al., 2013). Given the high risks associated with the Internet, parents have traditionally monitored their adolescents’ Internet use, such as checking adolescents’ whereabouts when using the Internet, setting rules on use, limiting time spent on the Internet, and preventing game playing (Liau et al., 2008). Indeed, parents have an important influence in monitoring and advising adolescents on Internet use to decrease adolescent’ risk of developing Internet gaming disorder (Lee and Chae, 2007; Bleakley et al., 2016). A meta-analysis of 57 studies conducted by Collier et al. (2016) found that parental monitoring is associated with decreased gaming time and sexual behavior in adolescents. Furthermore, a survey of 1800 adolescents in South Korea revealed that parental monitoring might be effective in reducing problematic mobile game use (Jang and Ryu, 2016). However, longitudinal data on how parental monitoring contributes to Internet gaming disorder in adolescents is inadequate.

Moreover, parents might practice less monitoring when they feel that their adolescents can control their online activities (Padilla-Walker and Coyne, 2011), which implies that the causality between parental monitoring and Internet gaming disorder might go in both directions. However, no longitudinal research has explored the reciprocal relationship between parental monitoring and Internet game disorder. Therefore, an in-depth understanding of the potential bidirectional relationship between parental monitoring and Internet gaming disorder is needed.

### The Role of Parent–Child Relationship in the Relation between Parental Monitoring and Internet Gaming Disorder

The quality of the parent–child relationship might underlie the bidirectional association between parental monitoring and Internet gaming disorder. In other words, parental monitoring could exert an effect on Internet gaming disorder via the parent–child relationship. According to the theoretical framework of self-determination theory, autonomy, competence, and relatedness are the three basic psychological needs that underlie intrinsic motivation in both children and adolescents (Ryan and Deci, 2000; Ryan et al., 2006). Adolescence is a period characterized by the seeking of greater autonomy and competence in decision-making, and parental monitoring might decrease adolescents’ opportunities to openly disclose information to their parents, thereby increasing the risk of negative outcomes (Kerr et al., 2010). In other words, a high level of parental monitoring might prevent children from satisfying their basic psychological needs (e.g., autonomy, competence, and relatedness), generating negative consequences such as parent–child conflict and hostility toward the parents, and leading to a poorer relationship (Kwon et al., 2011; Benrazavi et al., 2015; Su et al., 2015). A poorer parent–child relationship might also frustrate adolescents in satisfying their psychological needs, causing them to Internet gaming to meet their psychological needs when they feel uncomfortable with and misunderstood by their parents (Joussemet et al., 2008; Xu et al., 2012). Some studies have documented that the appeal of Internet games is based on satisfaction of individuals’ basic psychological needs (Przybylski et al., 2010), and adolescents might be more intrinsically motivated by the enjoyment of or interest in Internet gaming rather than by its outcomes because it satisfies their basic psychological needs (Ryan and Deci, 2000). A 3-year longitudinal study revealed that high parent–child relationships might lead to a decrease in the risk of Internet gaming disorder (Van den Eijnden et al., 2010). Similarly, findings from both European and Asian samples of adolescents have noted that high parent–child relationship is vitally important to the success of parental monitoring and the prevention of Internet gaming disorder (Kwon et al., 2011; Liu et al., 2013; Zhu et al., 2015; Bleakley et al., 2016; Wartberg et al., 2017). Given that the parent–child relationship is associated with both parental monitoring and Internet gaming disorder, it might have a mediating effect on the association between these two variables. However, no studies have yet examined such a mediating effect.

In addition, Videon (2005) suggested that fathers and mothers have unique impacts on different domains of child development because of their differing roles in most families. For example, mothers are more central to childrearing, parent–child communication, and emotional care, while fathers engage more actively in play and providing advice (LaRossa, 1985; Roopnarine and Mounts, 1985; Youniss and Smollar, 1985). Fathers and mothers also have differing effects on adolescents’ pathological Internet use (Liu et al., 2013) and problem behavior (Fosco et al., 2012). Still, little is known about the differing influences of mother– and father–child relationship on adolescents’ Internet gaming disorder.

### The Present Study

The present study examined the longitudinal reciprocal relationships among parental monitoring, parent–child relationship, and Internet gaming disorder in a Chinese sample using an autoregressive cross-lagged model. Although parental monitoring and parent–child relationship have been consistently found as correlates of Internet gaming disorder, we lack prospective evidence and an understanding of the mediating role of parent–child relationship. In this study, we specifically examine: (1) parental monitoring negatively predicts Internet gaming disorder; we also tested whether increased parental monitoring is a reaction to adolescents’ Internet gaming disorder. (2) Higher parental monitoring predicts significantly better father–child and mother–child relationships, which in turn predicts significantly lower Internet gaming disorder. (3) We tested the reverse direction of this previous set of relationships: that is, higher Internet gaming disorder predicts worse father–child and mother–child relationships, which in turn predicts lower parental monitoring.

## Materials and Methods

### Participants

Participants were recruited from three primary schools and two junior middle schools in China and were collected semiannually at three different points. At the baseline assessment (T1; October 2012), a total of 1830 adolescents were recruited. A total of 1680 of the adolescents recruited at T1 (91.80% of the original sample) participated in the second assessment (T2, April 2013) and 1490 (81.42% of the original sample) went on to complete the third assessment (T3, October 2013). Attrition was mainly due to the following reasons: (a) students were absent from school on the day of the assessment; (b) students had transferred to another school; (c) some students failed to answer most of the items; and (d) students refused to continue participating in the study. There were no significant differences in any of the studies variables between participants who completed all three assessments and participants with missing data.

The final sample of 1490 adolescents was 54.6% male and had a mean age of 12.03 years (*SD* = 1.59, range: 9–15 years) at T1; 692 were from primary schools (5th grade) and 798 were from junior middle schools (443 from 7th grade, 355 from 8th grade). About 41.3% were an only child, 92.5% came from intact families, and 7.5% came from single-parent families. Moreover, 22.3% of participants came from rural areas, 27.4% came from county seats, 41.9% came from small cities, and 8.3% came from large cities. Furthermore, 73.4% of participants’ mothers’ and 70.4% of their fathers’ education level was high school or less, and 52.2% of families had a per capita average monthly income of more than ¥ 3000, which is higher than the average in China in 2015 (Guangdong Statistical Yearbook, 2015; Zhu et al., 2017).

### Procedure

Trained teachers conducted a survey in participants’ classrooms using a standardized data collection process. At the beginning of each assessment, we obtained written consent informs from the teachers of the participating schools as well as participants and their parents. On the day of the assessment, students were invited to participate in their classrooms during class time. The trained teachers explained all the requirements of the assessment by reading the standardized instructions aloud. They also emphasized that all participants should complete the questionnaires honestly and independently without talking about their responses with any of their peers. During the assessment, all participants were told that their responses would be kept strictly confidential and that their participation was voluntary. Each collection took about 40 min. Ethics approval for this study was obtained from the South China Normal University Human Investigation Committee. Written informed consent was obtained from the teachers, all adult participants, and the parents/legal guardians of all non-adult participants.

### Measures

#### Parental Monitoring

We assessed parental monitoring using the 10-item Chinese version of the parental monitoring of Internet use questionnaire (Su et al., 2015), which was adapted from instruments used in previous studies (Liau et al., 2008). Adolescents were asked to indicate whether their parents were aware of their whereabouts during and the frequency, duration, accompanying peers, and contents of their Internet use in the past 6 months. Sample items include “My parents know where I’m surfing the Internet” and “My parents restrict me to using the Internet only at home.” All items were rated on a three-point scale (1 = never to 3 = often). We calculated the average of the 10-items, with higher scores indicating a greater level of parental monitoring. This questionnaire had Cronbach’s alpha values of 0.84, 0.87, and 0.86 at T1, T2, and T3, respectively.

#### Internet Gaming Disorder

The eight-item Internet Addiction Scale (IAS; Young, 1998) was used to measure adolescents’ Internet gaming disorder. This scale is rooted in the Diagnostic and Statistical Manual of Mental Disorders, Fourth Edition (DSM-IV) criteria for pathological gambling, and has been widely used to measure Internet gaming disorder (Gentile, 2009; Kwon et al., 2011; Yu et al., 2015; Zhu et al., 2015). Participants were queried about their degree of participation in Internet gaming, including “Do you feel upset or anxious when you try to cut down the amount of time you spend playing the Internet game?” and “Do you feel that you need to spent more and more time on online games?” All items were rated on a three-point scale (1 = never to 3 = often). We calculated the average of the eight-item scores, with higher scores reflecting a higher risk of Internet gaming disorder. The IAS had Cronbach’s alpha values of 0.76, 0.81, and 0.86 for T1, T2, and T3, respectively.

#### Parental–Child Relationship

This was measured using the 16-item Chinese version of the parent–child relationship questionnaire by Zhu et al. (2015). The internal consistency of this scale has been reported in prior researches to be greater than 0.80, which is adequate (Stattin and Kerr, 2000; Su et al., 2015; Zhu et al., 2015). Adolescents are asked about their feelings and conceptions over the past 6 months relating to their fathers and mothers (eight-items each). Sample items include “How often do you feel disappointed with your father?” “Have you quarreled with your father?” and “Do you feel proud of your father?” The eight-items on the mother–child relationship are the same as those for the father–child relationship. All items are rated on a three-point scale (1 = never to 3 = often). The average scores of the mother–child and father–child subscales are calculated separately, with higher scores indicating better father/mother–child relationship. The questionnaire had Cronbach’s alpha values of 0.78, 0.81, and 0.76 at T1, T2, and T3, respectively.

### Statistical Approach

First, descriptive statistics for all study variables and bivariate correlations were carried out in SPSS Statistics 20.0. Second, a longitudinal path analysis was conducted to examine the reciprocal effects between parental monitoring and Internet gaming disorder. Finally, we constructed separate autoregressive cross-lagged models for each type of parent–child relationship to examine its mediating effect on both directions of the path connecting parental monitoring and Internet gaming disorder. We followed the approach recommended by Cole and Maxwell (2003) to examine the autoregressive cross-lagged model with Mplus Version 7.0. Missing data were handled using the full information maximum likelihood estimation method. The bootstrap method was used to calculate the indirect effects (with 1,000 bootstrap samples). Good model fit was defined using the following criteria: χ^2^/df ≤ 3, comparative fit index (CFI) > 0.95, root mean square error of approximation (RMSEA) ≤ 0.06 (Hoyle, 2012). Based on prior findings, we controlled for several variables, such as gender, age, and family socioeconomic status (Collier et al., 2016; Mihara and Higuchi, 2017).

## Results

### Descriptive Statistics

**Table 1** reports the means, standard deviations, and correlations for the major study variables at each of the three waves of data. The results indicated that the bivariate correlations of the major variables in our hypothesized cross-lagged models were significant. Both parental monitoring and parent–child relationship at T1–T3 were negatively correlated with Internet gaming disorder at T1–T3. Parental monitoring at T1 was also positively correlated with father–child and mother–child relationship at T1–T3.

**Table 1:** Descriptive statistics and correlations for all variables (*N* = 1490).

Variable	1	2	3	4	5	6	7	8	9	10	11	12
(1) PM-T1	1											
(2) PM-T2	0.544**	1										
(3) PM-T3	0.431**	0.566**	1									
(4) FC-T1	0.208**	0.168**	0.152**	1								
(5) FC-T2	0.139**	0.214**	0.190**	0.425**	1							
(6) FC-T3	0.118**	0.124**	0.205**	0.388**	0.506**	1						
(7) MC-T1	0.194**	0.205**	0.155**	0.603**	0.269**	0.223**	1					
(8) MC-T2	0.207**	0.263**	0.219**	0.303**	0.611**	0.349**	0.418**	1				
(9) MC-T3	0.129**	0.132**	0.203**	0.234**	0.336**	0.609**	0.315**	0.441**	1			
(10) IGD-T1	-0.064*	-0.027	-0.060*	-0.173**	-0.183**	-0.160**	-0.167**	-0.176**	-0.171**	1		
(11) IGD-T2	-0.085**	-0.053*	-0.096**	-0.177**	-0.232**	-0.205**	-0.172**	-0.248**	-0.233**	0.519**	1	
(12) IGD-T3	-0.043	-0.055*	-0.084**	-0.101**	-0.205**	-0.242**	-0.109**	-0.189**	-0.255**	0.417**	0.574**	1
*M*	2.331	2.401	2.436	1.641	1.645	1.593	1.663	1.661	1.610	1.273	1.285	1.274
*SD*	0.513	0.524	0.501	0.184	0.190	0.196	0.186	0.195	0.201	0.298	0.325	0.355

*PM, parental monitoring; FC, father–child relationship; MC, mother–child relationship; IGD, Internet gaming disorder*. *^∗^p < 0.05 and ^∗∗^p < 0.01.*

### The Reciprocal Effects between Parental Monitoring and Internet Gaming Disorder

**FIGURE 1:**
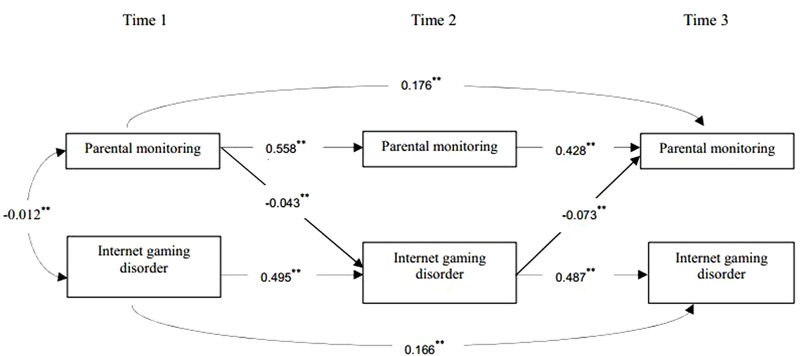
Cross- lagged structural equation model on the reciprocal effects between parental monitoring and Internet gaming disorder. Only significant paths are shown. Age, gender, and socioeconomic status were included as covariates in the model, but not displayed for model simplification. ^∗^*p* < 0.05 and ^∗∗^*p* < 0.01.

To examine the reciprocal effects between parental monitoring and Internet gaming disorder, we conducted and compared three parallel autoregressive models. Model 1 included the auto-regressive paths and concurrent correlations, and indicated a good fit to the data, χ^2^(6) = 2.872; CFI = 0.996, and RMSEA = 0.035. Then, Model 2 added the lagged paths from parental monitoring at T1 to Internet gaming disorder at T2 and parental monitoring at T2 to Internet gaming disorder at T3, as well as the path from Internet gaming disorder at T1 to parental monitoring at T2 and Internet gaming disorder at T2 to parental monitoring at T3. Model 2 proved a better fit than the baseline model (Model 1), χ^2^(2) = 0.255; CFI = 0.997, and RMSEA = 0.000, Δχ^2^(4) = 2.617, *p* > 0.05. Then, the path from parental monitoring at T1 to Internet gaming disorder at T3 and the path from Internet gaming disorder at T1 to parental monitoring at T3 were added, producing Model 3; however, Model 3 was a saturated model and the paths added to it were not significant. Thus, Model 2 was identified as the final model (see **Figure 1**).

The analyses of Model 2 indicated a reciprocal relationship between parental monitoring and Internet gaming disorder, which showed that greater parental monitoring at T1 significantly predicted less Internet gaming disorder at T2, *B* = -0.043, β = -0.069, *p* < 0.01, 95%CI [-0.067, -0.020]. Furthermore, Internet gaming disorder at T2 negatively predicted parental monitoring at T3, *B =* -0.073, β = -0.048, *p* < 0.01, 95%CI [-0.137, -0.019]. However, the path from parental monitoring at T2 to Internet gaming disorder at T3, as well as the path from Internet gaming disorder at T1 to parental monitoring at T2, were not significant.

### The Reciprocal Indirect Effects of Parent–Child Relationship on the Association between Parental Monitoring and Internet Gaming Disorder

**FIGURE 2:**
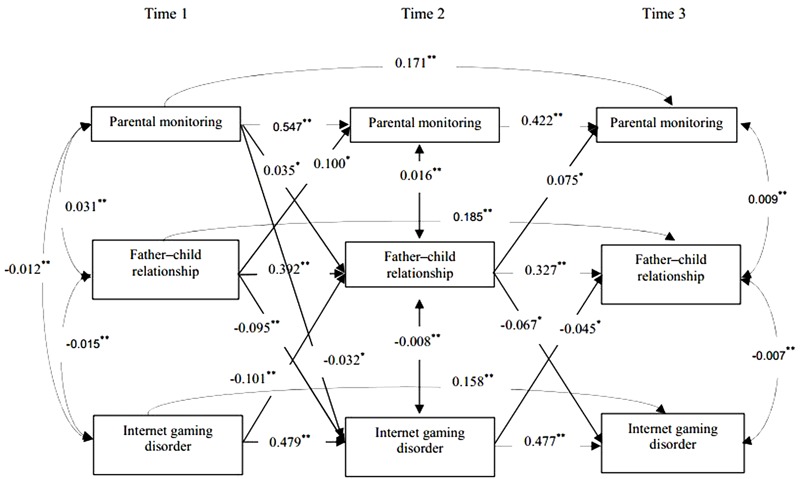
Cross-lagged structural equation model on the indirect effect of father–child relationship on the reciprocal connections between parental monitoring and Internet gaming disorder. Only significant paths are shown. Age, gender, and socioeconomic status are included as covariates in the model, but are not displayed for model simplification. ^∗^*p* < 0.05 and ^∗∗^*p* < 0.01.

**FIGURE 3:**
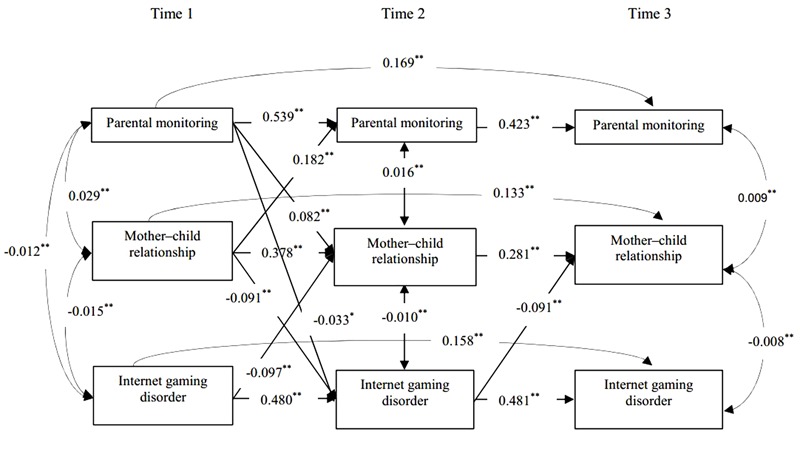
Cross-lagged structural equation model on the indirect effect of mother–child relationship in the reciprocal connections between parental monitoring and Internet gaming disorder. Only significant paths are shown. Age, gender, and socioeconomic status are included as covariates in the model, but are not displayed for model simplification. ^∗^*p* < 0.05 and ^∗∗^*p* < 0.01.

Based on the suggestion that a significant relationship between a predictor and a dependent variable is not a necessary condition to examine a mediating effect (Hayes, 2009), we tested the cross-lagged model using the stepwise method recommended by Cole and Maxwell (2003) to examine the mediating effect of parent–child relationship on the reciprocal association between parental monitoring and Internet gaming disorder. We first examine the mediating role of father–child relationship. At step 1, we ran Model 4-1, which contained autoregressive paths between parental monitoring, father–child relationship, and Internet gaming disorder, as well as concurrent covariances. Model 4-1 demonstrated a good fit to the data, χ^2^(18) = 4.994, CFI = 0.981, RMSEA = 0.051. At step 2, lagged paths between parental monitoring and Internet gaming disorder over time were added in Model 5-1, which showed a good fit to the data, χ^2^(14) = 5.135, CFI = 0.984, RMSEA = 0.053, Δχ^2^(4) = 0.141, *p* > 0.05. At step 3, the lagged paths between parental monitoring and father–child relationship, as well as the lagged paths between father–child relationship and Internet gaming disorder, were added in Model 6-1, which showed a good fit to the data, χ^2^(6) = 0.944, CFI = 1.000, RMSEA = 0.000, Δχ^2^(8) = 4.194, *p* > 0.05. At step 4, the path from parental monitoring at T1 to Internet gaming disorder at T3 and the path from Internet gaming disorder at T1 to parental monitoring at T3 were added in Model 7-1. Model 7-1, however, was a saturated model and the paths added in it were not significant. Thus, Model 6-1 was identified as the final model (see **Figure 2**). We followed similar steps to examine the mediating role of mother–child relationship; the final model (Model 6-2, see **Figure 3**) provided a good fit to the data, χ^2^(6) = 1.198, CFI = 1.000, RMSEA = 0.012, Δχ^2^(8) = 7.922, *p* > 0.05.

As shown in **Figure 2**, the path from parental monitoring at T1 to father–child relationship at T2 was significant, *B =* -0.031, β = 0.211, *p* < 0.01, 95% CI [0.024, 0.038]. In turn, father–child relationship at T2 negatively predicted Internet gaming disorder at T3, *B* = -0.008, β = -0.119, *p* < 0.01, 95%CI [-0.012, -0.005] (see central downward paths in **Figure 2**). Bootstrapping analyses indicated that the indirect effect of parental monitoring on Internet gaming disorder via father–child relationship was significant and positive, *B =* -0.002, *SE* = 0.002, β = -0.003, *p* < 0.05, 95% CI [-0.005, -0.001]. Meanwhile, Internet gaming disorder at T1 significantly predicted poorer father–child relationship at T2, *B* = -0.101, β = -0.102, *p* < 0.01, 95% CI [-0.149, -0.061]. In turn, poorer father–child relationship at T2 might decrease parental monitoring at T3, *B* = -0.067, β = -0.057, *p* < 0.05, 95% CI [-0.114, -0.022] (see central upward paths in **Figure 2**). Bootstrapping analyses indicated that the indirect effect of Internet gaming disorder on parental monitoring via father–child relationship was significant and positive, *B* = -0.008, *SE* = 0.003, β = -0.005, *p* < 0.05 and 95% CI [-0.017, -0.002].

As showed in **Figure 3**, parental monitoring at T1 predicted lower mother–child relationship at T2, *B =* 0.082, β = 0.140, *p* < 0.01, 95% CI [0.058, 0.106]. However, mother–child relationship at T2 did not predict Internet gaming disorder at T3, *B* = 0.058, β = 0.036, *p* > 0.05, 95% CI [-0.005, 0.121]. Meanwhile, adolescents’ Internet gaming disorder at T1 significantly predicted poorer mother–child relationship at T2, *B* = -0.097, β = -0.096, *p* < 0.01, 95% CI [-0.142, -0.058], but mother–child relationship at T2 did not predict parental monitoring at T3, *B* = -0.042, β = -0.037, *p* < 0.05, 95% CI [-0.085, 0.001] (see central upward paths in **Figure 3**). A significant indirect effect of parental monitoring on Internet gaming disorder via mother–child relationship was not found.

## Discussion

Several important findings emerged from this longitudinal study that may enhance our understanding of the associations between parental monitoring, Internet gaming disorder, and parent–child relationship. First, we found a reciprocal relationship between parental monitoring and Internet gaming disorder. Second, father–child relationship has a mediating effect on both directions of the path between parental monitoring and Internet gaming disorder; however, significant indirect effects of parental monitoring on Internet gaming disorder (and vice versa) via mother–child relationship were not found, indicating that there were different impacts of father–child and mother–child relationship on the reciprocal relationship between parental monitoring and Internet gaming disorder. Altogether, the findings suggest that parental factors (e.g., higher parental monitoring of Internet use and better father–child relationship) likely contribute to decreased risk of Internet gaming disorder in early adolescence.

### Direct Effects

The results indicate that parental monitoring negatively predicts adolescents’ Internet gaming disorder. However, the relationship between parental monitoring and Internet gaming disorder in this study was not stable over time. In fact, parental monitoring at T1 predicted lower levels of Internet gaming disorder at T2, but parental monitoring at T2 did not significantly predict Internet gaming disorder at T3. This finding suggests an inconsistency in the effects of parental monitoring on Internet gaming disorder. Consistent with our findings, some earlier studies have shown that a higher level of parental monitoring was effective for reducing problematic Internet use and other behavioral problem (e.g., Bleakley et al., 2016; Collier et al., 2016; Jang and Ryu, 2016). However, some empirical studies have indicated that parental monitoring, such as restriction of video gaming, might not be an effective method of reducing Internet gaming disorder (Livingstone and Helsper, 2008; Van den Eijnden et al., 2010; Shin and Huh, 2011; Choo et al., 2015). One possible explanation might be the different parenting styles between Chinese culture and Western culture. Western parents are more respectful of children’s personal choices and self-esteem, whereas Chinese parents tend to emphasize parental authority and more readily supervise their children’s behaviors (Chao and Tseng, 2002). Therefore, Chinese parents employ more monitoring and restriction of their children’s behaviors when adolescents show problem behaviors such as excessive Internet use, whereas Western parents tend to increase parent–child communication, and provide more advice and support in helping their adolescents set rules (Su et al., 2015; You et al., 2017). Moreover, Chinese adolescents who grow up in this traditional culture are more likely to accept their parents’ monitoring than are Western adolescents.

The results of the path from Internet gaming disorder to parental monitoring indicated that Internet gaming disorder at T2 was significantly associated with parental monitoring at T3. The present study indicate that when parents are aware that their adolescents are at risk of Internet gaming disorder, they appear to engage in less monitoring of their children’s behaviors after half a year. Contrary to this finding, previous studies found that parental monitoring was associated with recognition of adolescents’ self-regulation ability: that is, when parents perceive that adolescents cannot control their online activities, they tend to practice more monitoring (Padilla-Walker and Coyne, 2011). One possible explanation for this discrepancy is that parents tend to respond to Internet gaming disorder by immediately increasing harsh monitoring, but after half a year, when they feel that such harsh monitoring cannot successfully control their adolescents’ online gaming activities, they might ease up on their monitoring (Su et al., 2015). Studies on adolescents’ externalizing problems have also found that the failure in perceived parental control attempts might lead to a reduction in subsequent parental control attempts (Pinquart, 2017). Another possible explanation is that adolescents with high levels of Internet gaming disorder might learn how to adapt to parental monitoring. Problematic gamers experiencing exaggerated gaming motivation might devise creative ways to engage in their activity of interest. In other words, they might initially play less because of parental monitoring, while working to procure ways of engaging in the activity that avoids parental notice (e.g., playing at friend’s house, saving money to buy their own device and hiding it, skipping class or other activities to go to gaming centers). Furthermore, parents are less able to monitor adolescents as they age because of the increasing amount of time adolescents spend outside the home. Additionally, one study found that around 49% of adolescent aged 9–16 used the Internet in their bedrooms, which makes it harder for parents to monitor even though adolescents are still using the Internet at home (Livingstone et al., 2011). Considering the direct effect between parental monitoring and Internet gaming disorder in this study was not stable over time, it should be interpreted with caution until there is more in-depth research and adequate evidence.

### Longitudinal Indirect Effects

We believe that the most important finding in this study is that the father–child and mother–child relationships have different influences on the reciprocal relationship between parental monitoring and adolescent Internet gaming disorder. Specifically, only father–child relationship had a reciprocal indirect effect on the relationship between parental monitoring and Internet gaming disorder; no associations were found for mother–child relationship. Although many studies have been reported the importance of mother–child relationship, the present study revealed that fathers might be more important in preventing adolescents’ Internet gaming disorder. These findings accord with those of another longitudinal study showing that parental monitoring and father–child connectedness are associated with reductions in problem behavior in a sample of 6th to 8th grade students over time (Fosco et al., 2012). Furthermore, Liu et al. (2013) found that father–child relationship predicted Internet gaming disorder while perceived mother–child relationship did not. One possible explanation is that fathers and mothers play different roles in most families, and thus have different ways of influencing their adolescents. Prior literature has shown that mothers specialize in the expressive role (e.g., childrearing, communication, and emotional care), whereas fathers adopt an instrumental role (e.g., financial support, play, providing advice and guidance; Youniss and Smollar, 1985; Videon, 2005). Therefore, mothers might have a greater influence on adolescents’ emotion, whereas fathers might mainly influence adolescents’ behavior (Fosco et al., 2012; Liu et al., 2013; Feld, 2015; Pinquart, 2017). In this case, fathers might play a crucial role when adolescents begin to develop social skills and look for guidance in handling difficult situations. Adolescents are known to be harmed by their fathers’ absence due to a lack of involvement and sufficient support, and as a result are more likely to seek an online activity (Videon, 2005; Su et al., 2015). Another explanation might be cultural differences in parenting and parent–child relationships. In Chinese culture, fathers are regarded as indifferent dominators at the center of a family, and tend to scold and criticize their adolescents more in an effort to rule adolescents’ behaviors (You et al., 2017). Adolescents who feel alienated from their fathers are more inclined to feel rejected and might more readily seek connections with video games (Liu et al., 2013).

Analyses of the reverse direction in our cross-lagged model also suggests that high levels of Internet gaming disorder might initially lead to poorer father– and mother–child relationships, but then only poorer father–child relationship led to higher parental monitoring. Adolescents who are addicted to video games might cause parents anxiety in relation to the consequences of Internet gaming disorder, such as academic and behavioral problems (Young and de Abreu, 2011; Griffiths et al., 2015). Consequently, parents tend to be more critical of them, which might lead to worse parent–child conflict and subsequent a worse parent–child relationship. As discussed above, fathers tend to be more involved in correcting children’s problematic behavior, whereas mothers are more involved in emotional care. Therefore, adolescents who experience higher Internet gaming disorder and poorer father–child relationships are more likely to monitor Internet use.

Although the role of parent–child relationship was the primary focus of the present study, the findings revealed the further possibility that Internet gaming disorder can act as a mediator of the relationships between parental monitoring and parent–child relationship. Specifically, the cross-lagged model indicated that high parental monitoring of Internet use might lead to a decrease in Internet gaming disorder, which might then lead to higher quality parent–child relationships.

### Implications

To our knowledge, the present study is the first to test the reciprocal relationships between parental monitoring, parent–child relationship, and Internet gaming disorder. The findings of this study might have implications for targeted interventions. Particularly, parental monitoring was found to be a direct predictor of Internet gaming disorder in this study. Therefore, parents’ behaviors in managing adolescents’ Internet gaming disorder might be important. This study provides evidence to support the suggestion that general parental monitoring, including obtaining more knowledge about daily adolescent activities and setting rules about Internet use time, place, and content, might decrease adolescents’ Internet gaming disorder. For effective parental monitoring of adolescent Internet use, the best strategy might be to pursue open communication with adolescents, as well as build up connections between parents and their adolescents while considering their Internet use (Symons et al., 2017). Second, we examined the potential differences in the impact of father–child and mother–child relationship on the bidirectional association between parental monitoring and Internet gaming disorder in the current study. According to our results, and taking into consideration previous reports on the family-based approach (Lochman and Van den Steenhoven, 2006; Liu et al., 2012), fathers seem to be more important in preventing Internet gaming disorder over time. This finding highlights the unique contributions of fathers in attending to their adolescents’ activities and providing rules and supervision on adolescents’ Internet use; meanwhile mothers contribute on emotional warm and communications.

### Limitations

There are several limitations in the current study. First, because all measures in this study were collected from adolescents’ self-report, not from their parents, the findings might be somewhat limited because of common method variance and social desirability bias. Studies have indicated there might be considerable differences between adolescents’ self-report and parental reports about adolescents’ Internet use and parental monitoring: namely, 62% of parents reported that they have supervised their adolescents’ online activities, but only 33% of adolescents reported believing that parents have correct knowledge of their Internet use (Liau et al., 2008). Second, the study was conducted in a Chinese cultural background with a convenience sample, so the generalizability of the findings is potentially low. As noted previously in our discussion, there might be differences in parenting style between different culture backgrounds. Future research should verify these findings in a large representative sample in other cultures. Third, we should be cautious in extending our results to other types of parenting behavior, such as parental knowledge and parental mediation strategies (including restrictive mediation, active mediation, and co-playing), although they might be similar to each other (Livingstone and Helsper, 2008; Bleakley et al., 2016). Prior literature has noted that the inconsistence in parental monitoring effects might be due to the definitions and measurement tools used, particularly the medium, content, or outcome analyzed by these tools (Collier et al., 2016). For example, some argue parental monitoring is actually a measure of “parental knowledge” (Stattin and Kerr, 2000) and others suppose that restrictive mediation might be viewed as a form of parental monitoring (Collier et al., 2016). Future research should explore these potential monitoring effects in greater depth. Fourth, despite the existence of a precise definition and diagnostic criteria for Internet gaming disorder, there are a multitude of measuring tools, none of which have achieved global consensus. Although Young’s eight-item IAS is widely used for both screening and diagnostic purposes, as well as for estimating the prevalence of Internet gaming disorder in epidemiological studies (e.g., Yu et al., 2015; Zhu et al., 2015; Mihara and Higuchi, 2017), some studies have questioned the appropriateness of measures for Internet gaming that are based on measures of pathological gambling, as they might be inaccurate or inapplicable to all people or situations (e.g., Ko et al., 2014; Petry et al., 2014; Laconi et al., 2017). Future research should use consistent methods of measuring Internet gaming disorder based on the nine criteria of the DSM-5.

## Conclusion

Our findings expand on previous studies suggesting that parents play a vital role in preventing Internet gaming disorder among adolescents. A reciprocal direct relationship between parental monitoring and Internet gaming disorder was found—that is, parental monitoring at T1 predicted lower Internet gaming disorder at T2 and a high level of Internet gaming disorder at T2 predicted less parental monitoring at T3. A reciprocal, indirect effect of father–child relationship on the link between parental monitoring and Internet gaming disorder was also found, but no such indirect effect was found for mother–child relationship. Our findings call for further longitudinal studies using more consistent methods of measuring Internet gaming disorder and parental monitoring, as well as alternative methods of data collection (e.g., interviews with parents or Internet gamers). This would aid in our understanding of how parental factors influence Internet gaming disorder in different cultural and social environments.

## Author Contributions

Conceived and designed the research: BS and WZ. Performed the research: BS, CY, and JZ. Analyzed the data: BS, QS, CY, and YJ. Contributed to the writing of the manuscript: BS, CY, WZ, QS, JZ, and YJ.

## Conflict of Interest Statement

The authors declare that the research was conducted in the absence of any commercial or financial relationships that could be construed as a potential conflict of interest.
